# Chemosensation of the pheromone spermine by the olfactory TAAR-like receptor TAAR348

**DOI:** 10.1038/s41421-025-00839-4

**Published:** 2025-09-27

**Authors:** Kexin Jiang, Liting Zeng, Weifeng Zhang, Xuefei He, You Zheng, Ling Wang, Zhongyuan Zhang, Jun Pu, Cheng Deng, Fan Yang, Suwen Zhao, Fei Xu

**Affiliations:** 1 iHuman Institute, School of Life Science and Technology, ShanghaiTech University, Shanghai, China; 2 Department of Physiology and Pathophysiology, School of Basic Medical Sciences, Shandong University, Jinan, Shandong China; 3 Department of Respiratory and Critical Care Medicine, Center for High Altitude Medicine, National Clinical Research Center for Geriatrics, West China Hospital, Sichuan University, Chengdu, Sichuan China; 4 Department of Neurosurgery, The Second Affiliated Hospital of Kunming Medical University, Kunming, Yunnan China

**Keywords:** Cryoelectron microscopy, Cell signalling

Dear Editor,

Trace amine-associated receptors (TAARs) are a vertebrate-specific subfamily of G protein-coupled receptors (GPCRs) that are broadly expressed in the mammalian central nervous system and involved in olfaction, behavior, and emotional regulation^1^. Since their initial cloning in 2001, TAARs have been identified across diverse species, exhibiting notable evolutionary diversity^2^. The TAAR family can be divided into three major evolutionary clades: Clade I consists of conserved single-copy genes; Clade II shows species-specific expansions; and Clade III is primarily found in teleosts^3^. Additionally, a group of TAAR-like genes, although evolutionarily distinct from canonical TAARs, retains chemosensory functions^4^ (Fig. 1a; Supplementary Fig. S1a). Recent structural elucidation of TAAR1^5,6^, TAAR9^7^, and TAAR7f^8^ has significantly advanced our understanding of ligand recognition mechanisms in the TAAR family. This advancement is particularly important for guiding drug discovery efforts targeting this unique class of aminergic receptors, which are significantly involved in a range of behavioral and neurological disorders. However, structural studies on the more abundant olfactory TAAR-like receptors remain limited, and their ligand recognition mechanisms and structural features are still poorly understood.

**Fig. 1:**
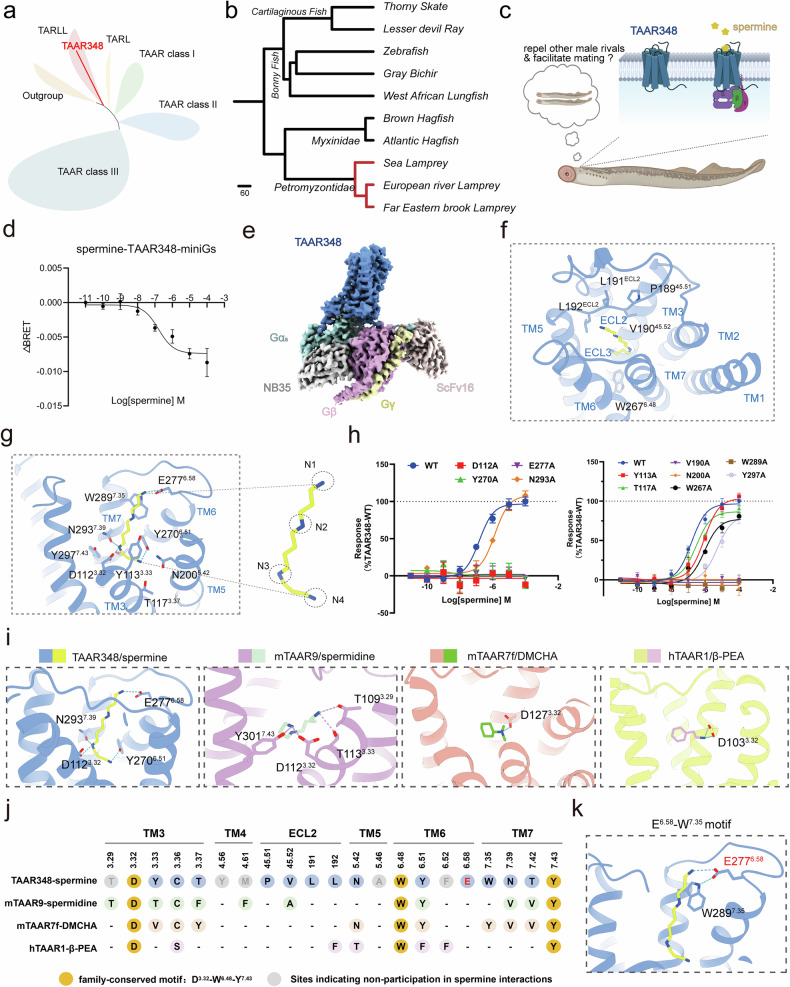
Structural and functional characterization of spermine recognition by TAAR348. **a** The TAAR family is divided into distinct classes based on phylogenetic analysis. TAAR348 belongs to the TAAR-like branch in sea lampreys. **b** Phylogenetic tree depicting the relationships among a total of 10 species, including both jawed and jawless vertebrates. Red line indicates the nodes containing TAAR348 and its orthologs. Branch lengths indicate divergence time (million years ago, MYA). **c** Conceptual model of spermine sensing by TAAR348 in sea lampreys, highlighting the potential role of TAAR348 in modulating behaviors through spermine sensation. Figure created with http://BioRender.com. **d** The dose-response curve illustrates the BRET2 assay results for spermine activating TAAR348, conducted in HEK293 cells overexpressing TAAR348 and G_s_ sensors. **e** Cryo-EM density map of the spermine–TAAR348–G_s_ complex. The complex density map is shown at a contour level of 0.14. Blue, spermine-bound TAAR348; green, Gα_s_; pink, Gβ; yellow, Gγ; gray, NB35; orange, ScFv16. **f** The non-polar interactions between spermine and residues from TAAR348. **g** Key polar interactions between spermine and residues from TAAR348. The hydrogen-bonding network to stabilize the interaction is depicted. Sequential designation of the four amine groups of spermine as N1 to N4. **h** BRET ratios measured for increasing concentrations of the agonist spermine for the wild-type TAAR348 (WT) and 11 mutants, and normalized with respect to cell surface expression levels. Experiments were performed independently three times (see Supplementary Table S3 for details), with a single measurement per experiment; error bars represent the SEM. Each mutant response was normalized to the corresponding TAAR348 (WT), which was set to 100%. **i** Structural comparison highlighting key hydrogen bonds and salt bridges between the amine groups of the ligand and receptor residues in TAAR348–spermine, mTAAR7f–DMCHA, mTAAR9–spermidine, and hTAAR1–β-PEA complexes. The conserved salt bridge formed by D^3.32^ with the central amine is shown in all complexes. In the spermine–TAAR348 complex, the terminal amines N1 and N4 also form key interactions, further stabilizing spermine’s extended conformation. Interactions are depicted as dashed lines. **j** Barcode representation of interaction patterns in the ligand pockets of TAAR348–spermine, mTAAR7f–DMCHA, mTAAR9–spermidine, and hTAAR1–β-PEA complexes. The conserved residues forming the generic motif for amine recognition across the three complexes are indicated by orange circles. Residues exhibiting no interaction with the respective ligands are shown as gray circles. **k** Structural representation of the spermine–TAAR348 interactions highlighting the critical role of the E^6.58^-W^7.35^ motif in ligand recognition.

As one of the most ancient extant vertebrates, the sea lamprey possesses a highly sensitive olfactory system, making it a key model for studying the evolution of TAARs^9^. Phylogenetic analyses demonstrate that TAAR348 is uniquely restricted to the petromyzontidae lineage, with its genomic locus exhibiting strong colinearity across species within this lineage (Fig. 1b; Supplementary Fig. S1b and Table S1), suggesting an evolutionarily constrained function. Here, we focus on the structurally uncharacterized but functionally pivotal TAAR-like receptor, TAAR348, to uncover the molecular basis of chemosensation and its evolutionary significance within the TAAR family.

Although evolutionarily distinct from canonical olfactory receptors (ORs), TAARs represent the second-largest class of vertebrate olfactory GPCRs and are essential for odor detection^10^. A pioneering study revealed that spermine, a potent male sex pheromone present in the seminal fluid of sea lampreys, specifically activates TAAR348, thereby attracting sexually mature females and promoting mating and spawning behaviors^11^ (Fig. 1c). Beyond its pheromonal role, spermine is a biologically essential polyamine involved in spermatogenesis, testicular steroidogenesis, and core cellular processes including proliferation, survival, and growth.

In this study, we first confirmed that spermine can activate TAAR348’s G_s_ pathway, exhibiting an EC_50_ of 155.4 ± 6.9 nM (Fig. 1d), as measured by the G protein trimer dissociation assay (BRET2 assay, see Supplementary Materials and Methods). To elucidate the structural basis, we determined the cryo-EM structure of the spermine-bound TAAR348–G_s_ complex at a resolution of 2.82 Å (Fig. 1e; Supplementary Figs. S2, S3 and Table S2). The receptor adopts a canonical class A GPCR architecture with a seven-transmembrane helical bundle (Supplementary Fig. S4a–c). Spermine occupies the orthosteric binding site and forms a conserved salt bridge with D112^3.32^, a hallmark of aminergic receptors in recognition of amines (Supplementary Fig. S4e). TAAR348 shares close structural similarity with other TAARs^6–8^ (with RMSD ranging from 1.1 Å to 1.5 Å when aligned with the existing TAAR structures) but differs notably from OR51E2^12^ (the classical olfactory receptor, with an RMSD over 2 Å, Supplementary Fig. S4d). Its extended ligand-binding pocket accommodates the linear conformation of spermine, bridging ECL2 and ECL3 and aligning toward TM6 (Supplementary Fig. S4e, f), defining a distinct ligand-binding mode.

Further analysis shows spermine is stabilized in the TAAR348 orthosteric binding site by salt bridges, hydrogen bonds, polar networks, and hydrophobic interactions. The ligand adopts an “L”-shaped conformation within a transmembrane cavity formed by TM3, TM5, TM6, and TM7 (Fig. 1f; Supplementary Fig. S5a, b). Its upper segment extends between ECL2 and ECL3, making hydrophobic contacts with ECL2 residues P189, V190, L191 and L192, while the lower end interacts with W267^6.48^ (Fig. 1f). The pocket is predominantly negatively charged attributed to a cluster of aspartate and glutamate residues, resembling the polyamine-binding site of human ATP13A2^13^ (Supplementary Fig. S5a–d), though spermine assumes a more extended shape in ATP13A2. We propose that within the binding pocket of TAAR348, the terminal amine group of spermine forms polar interactions mainly with Y270^6.51^, while the bulky W267^6.48^ side chain restricts spermine’s downward extension by steric hindrance (Fig. 1f, g).

Notably, a coordinated network of residues anchors the four amine groups (N1 to N4) of spermine. N1 forms a salt bridge with E277^6.58^, which is further stabilized by W289^7.35^; N2 interacts with Y113^3.33^; N3 forms salt bridge and polar contacts with D112^3.32^ and N293^7.39^; and N4 engages Y270^6.51^, T117^3.37^, and N200^5.42^. Together, these interactions anchor spermine precisely within the TAAR348 pocket (Fig. 1g).

To assess the functional relevance of spermine-binding residues, we performed alanine mutagenesis followed by BRET2 assays. Substitutions at D112^3.32^, E277^6.58^, Y270^6.51^, N200^5.42^, W289^7.35^, and V190^45.52^ nearly abolished receptor activity, while mutations at Y113^3.33^, T117^3.37^, N293^7.39^, and Y297^7.43^ markedly reduced the potency of spermine-activated G_s_ signaling (Fig. 1h; Supplementary Fig. S8e and Table S3). These results underscore the essential role of salt bridges, hydrogen bonds, polar interactions, and hydrophobic contacts in TAAR348’s ligand recognition and activation.

TAAR348 is classified as a TAAR-like receptor, but its structural features remained unknown. To explore its relationship with canonical TAARs, we compared the ligand-binding pockets of TAAR348, mTAAR9^7^, TAAR7f^8^, and hTAAR1^5^. Structural alignment revealed that TAAR348 retains the conserved D^3.32^-W^6.48^-Y^7.43^ motif characteristic of the TAAR family. Spermine forms a canonical salt bridge with D^3.32^, indicating that TAAR348 follows the aminergic recognition pattern typical of TAARs (Fig. 1i, j).

A key feature of TAAR348 is the presence of E^6.58^, which directly contributes to spermine recognition and stabilizes its L-shaped conformation (Fig. 1g, j). In contrast, some TAARs (e.g., TAAR1, TAAR7f, TAAR9) have D^6.58^ at this position (Supplementary Fig. S8a). This difference may reflect adaptive evolution toward spermine recognition. Spermine and its precursor spermidine are structurally related polyamines but differ in chain length and amine group number^14^, resulting in distinct recognition mechanisms. While spermidine-activated mTAAR9 engages a T^3.29^-T^3.33^ and F^3.37^-F^4.61^-Y^6.51^-V^7.39^ motif, spermine recognition by TAAR348 relies on E^6.58^-W^7.35^ and the T117^3.37^-N200^5.42^-Y270^6.51^ motifs (Fig. 1j), which likely maintains the ligand’s L-shaped conformation. These findings suggest that TAAR348 preserves core aminergic features while evolving a distinct binding mode, offering insight into the structural and functional diversity of TAAR-like receptors.

Comparison of the spermine-bound TAAR348 structure with the active-state TAAR1 (PDB: 8W89)^5^ and inactive β2-adrenergic receptor (β2AR; PDB: 3NY8)^15^ reveals that TAAR348’s transmembrane helices closely resemble those of active TAAR1 (Supplementary Fig. S6a, b). Activation involves a hydrophobic interaction between spermine and the toggle-switch residue W267^6.48^, inducing a downward shift of the W267^6.48^ side chain (Supplementary Fig. S6c). This triggers conformational changes in R130^3.50^ of the DRY motif and F263^6.44^ of the PIF motif, along with an inward movement of TM7 in the NPxxF motif. Notably, Y^7.53^ in TAAR1 is replaced by F^7.53^ in TAAR348, and TM7’s inward shift is less pronounced (Supplementary Fig. S6d–f). These rearrangements disrupt the ionic lock and drive a marked outward displacement of the intracellular end of TM6 relative to inactive β2AR (Supplementary Fig. S6b). Overall, TAAR348 undergoes conformational rearrangements of key motifs during transition from inactive to active states, resembling the classical activation mode of class A GPCRs.

To elucidate the mechanism underlying polyamine recognition and family specificity of TAAR348, we combined molecular dynamics (MD) simulations, sequence alignment, evolutionary analysis, and functional assays. MD simulations demonstrated that spermine remains highly stable within the orthosteric binding site of TAAR348 (Supplementary Fig. S7a). In particular, N1 and N3 formed salt bridge and polar contacts with their paired residues at ~3 Å, while N4 maintained the distance with the surrounding residue at ~5 Å (Supplementary Fig. S7b). Critical residues for amine recognition were confirmed, with the conserved D^3.32^-W^6.48^-Y^7.43^ motif playing a central role (Fig. 1j; Supplementary Fig. S8a). A conserved polar network involving S^2.61^, R^2.64^, H^3.28^, D^3.32^, and Y^7.43^ — conserved across TAAR1, TAAR5, TAAR9, and TAAR7f — supports the structural integrity of the ligand-binding site (Supplementary Fig. S8a, c–e and Table S3). Of particular note is the unique E^6.58^-W^7.35^ motif in TAAR348, which highlights its key role in spermine recognition (Fig. 1k; Supplementary Fig. S8b, e and Table S3).

Given the limited understanding of TAAR-like receptors in early vertebrates, deciphering their ligand recognition mechanisms is essential for uncovering the functional evolution of the TAAR family. In this study, we report the first structural characterization of a TAAR-like receptor and reveal a multivalent recognition strategy in TAAR348 that differs markedly from the monoamine sensing typical of mammalian TAARs (Fig. 1g, i; Supplementary Fig. S8b). This suggests an adaptive mechanism for polyamine chemosensation, potentially underlying the ability of sea lampreys to sense spermine in aquatic environments. It also raises the possibility that similar cooperative binding modes may be employed by other GPCRs.

Beyond evolutionary insights, the molecular features of TAAR348 provide a promising scaffold for engineering spermine-specific biosensors with potential applications in environmental monitoring and disease diagnostics. TAAR348 thus serves as a valuable model for exploring ligand recognition, sensory adaptation, and signal transduction in basal vertebrates. Future studies will focus on expanding the structural and functional characterization of TAAR-like receptors across diverse species to better understand their evolutionary trajectories and physiological roles.

## Supplementary information

Supplementary Information

## Data Availability

The cryo-EM density maps and atomic coordinates for the spermine–TAAR348–G_s_ complex have been deposited in the Electron Microscopy Data Bank (EMDB) and Protein Data Bank (PDB), respectively, under accession numbers EMD-65187 and 9VMG.
