# Supplementary material for: Chemosensation of the pheromone spermine by the olfactory TAAR-like receptor TAAR348
Source: Cell Discov. 2025 Sep 27;11:79. doi: 10.1038/s41421-025-00839-4 (PMC12476453; doi:10.1038/s41421-025-00839-4)
Supplement: Supplementary file 1 — Supplementary Information [file 41421_2025_839_MOESM1_ESM.pdf]

Materials and Methods

Phylogenetic Analysis

The amino acid sequences of trace amine-associated receptors (TAARs) and other aminergic receptors were analyzed across multiple species, including human, mouse, rat, chimpanzee, zebrafish, and sea lamprey. A high-quality multiple sequence alignment (MSA) was generated using L-INS-i.[1] Phylogenetic tree construction was performed with IQ-TREE[2] using the JTT+F+R5 model with 1,000 ultrafast bootstrap replicates (-bb 1000), and other aminergic receptors were included as an outgroup. The tree was visualized with iTOL.[3]

TAAR annotation was performed across chordate 2666 species, resulting in the identification of 29,895 TAAR sequences. To reduce redundancy, CD-HIT[4] was used to cluster protein sequences at an 80% identity threshold, yielding a final dataset of 3,196 representative sequences. The known 153 TAAR sequences form UniProt[5] and published literature[6] were first aligned using L-INS-i[1] to generate a high-quality multiple sequence alignment (MSA). Then, the representative TAAR sequences were incorporated into this pre-aligned MSA using MAFFT[1] with the --keeplength and --add options, ensuring that the original alignment structure was preserved while minimizing the introduction of excessive gaps. This approach enabled the alignment of over 3,000 representative TAAR sequences efficiently. Phylogenetic tree construction was performed with IQ-TREE[2] using the JTT+F+R5 model with 1,000 ultrafast bootstrap replicates (-bb 1000), and other aminergic receptors were included as an outgroup.

A total of 136 TARLL sequences were identified from nine jawless fish species. All TARLL nucleotide sequences were aligned using L-INS-i,[1] and a maximum-likelihood (ML) tree was constructed using IQ-TREE[2] under the GTR+F+R10 model with 1,000 ultrafast bootstrap replicates (-bb 1000). The final tree was visualized using iTOL.[3]

Phylogenetic Tree Construction

To identify orthologues of TAAR348 in fish, we selected five jawless and five jawed fish species with chromosome-level reference genome assemblies (see Supplementary Table S1 for

species list and genome versions). The phylogenetic tree was constructed using divergence time data obtained from TimeTree 5,[7] and the Newick file was visualized and refined using FigTree v1.4.4.

Orthologues Alignment

The genomic sequence of *TAAR338/LOC116944353* (*NC_046089.1:11770433-11771476*) from the sea lamprey was obtained and used as a query for nucleotide-nucleotide BLAST 2.15.0+ against other species. The BLASTN parameters were set as follows: -evalue 1e-10 -perc_identity 60 -max_target_seqs 3. Only two species, *European river lamprey* and *Far Eastern brook lamprey*, exhibited orthologous segments under these criteria.

Pairwise Genome Alignment

Based on the results from BLASTN, we extracted chromosome sequences from three lamprey species where the target segments were located. Pairwise genome alignments (using sea lamprey as the query) were conducted using the LAST (Larger Acceptance Seed Threshold) v1452 toolkit, which includes last-train, lastal, maf-swap, last-split, and last-postmask. These tools facilitated the generation of one-to-one MAF (Multiple Alignment Format) results and synteny analysis. The R package "RIdeogram" was employed for visualizing the multiple alignment results.

Cell lines

Spodoptera frugiperda (Sf9, Expression systems) and *Trichoplusia ni.* (High Five, Thermo Fisher) cells were grown in ESF 921 medium at 27 °C and 120 rpm. HEK293T cells were grown in a humidified 37 °C incubator with 5% CO_2 using media supplemented with 100 I.U./mL penicillin and 100 mg/mL streptomycin (Invitrogen). The human cell lines HEK293T were maintained in DMEM (VWR) containing 10% fetal bovine serum (FBS, VWR).

Constructs

For structure determination of the spermine activated TAAR348-Gs complex, the wild type (WT) TAAR348 construct was cloned into the pFastBac1 vector with the N-terminal

haemagglutinin signal peptide (HA) followed by a Flag tag, 10×His tag and human rhinovirus 3C (HRV 3C) protease site. To enhance surface expression of the receptor, a thermostabilized Escherichia coli apocytochrome *b*562RIL (BRIL) fusion protein was fused at the N-terminus[8] of TAAR348 linked by the first nine amino acids of the β_2-adrenergic receptor (MGQPGNGSA, β9)[9]. An engineered mini-$G\alpha_s$ was fused to the C-terminus of TAAR348 with three copies of 3C protease sites between them. $G\beta_1$ and $G\gamma_2$ were cloned into a pFastBac Dual vector.

Purification of spermine bound TAAR348-Gs-ScFv16-NB35 complex

The complex is expressed in High Five insect cells (Thermo Fisher). For expression of TAAR348-Gs complex, cell cultures were grown in ESF 921 medium to a density of 3×10^6 per mL with two separate virus preparations for TAAR348-miniGαs and Gβ1γ2 at a ratio of 1:1.2. The infected cells were cultured at 27 °C for 48 h before collection by centrifugation and the cell pellets were stored at -80 °C for future use.

For the purification of the spermine-bound TAAR348-Gs complex, cell pellets from 1 L culture were thawed at room temperature and resuspended in low-salt buffer containing 20 mM HEPES (pH 7.4), 100 mM NaCl, 5 mM $CaCl_2$, 5 mM $MgCl_2$, 10% glycerol, and protease inhibitor cocktail (Thermo Fisher). The TAAR348-Gs complex was formed on the membrane in the presence of 500 μM spermine (Sigma) and treated with 20 mU mL^{-1} apyrase (NEB), followed by incubation overnight at 4 °C. Cell membranes were collected by ultracentrifugation at 119,000×g for 40 minutes. The membranes were then resuspended and incubated with 500 μM spermine, 2 mg mL^{-1} iodoacetamide (Sigma), and 20 mU mL^{-1} apyrase at 4 °C for 1 h. The protein was extracted from the membrane using a buffer containing 20 mM HEPES (pH 7.4), 100 mM NaCl, 5 mM $CaCl_2$, 5 mM $MgCl_2$, 10% glycerol, 1% (w/v) lauryl maltose neopentyl glycol (LMNG, Anatrace), and 0.2% (w/v) cholesterol hemisuccinate (CHS, Anatrace) and stirred for 2.5 hours at 4 °C. The supernatant was isolated by ultracentrifugation at 119,000×g for 40 minutes and then incubated overnight at 4 °C with pre-equilibrated TALON IMAC resin (Clontech). After batch binding, the TALON IMAC resin containing the immobilized protein complex was manually loaded onto a gravity flow column. The TALON IMAC resin was washed with 10 column volumes of buffer containing 20 mM HEPES (pH 7.4), 100 mM NaCl, 5 mM $CaCl_2$, 5 mM $MgCl_2$, 30 mM imidazole, 10% glycerol, 0.1% LMNG (w/v), 0.02% CHS

(w/v), and 500 μM spermine, and eluted with the same buffer plus 300 mM imidazole and 1 mM spermine. The eluted protein was incubated with 20 μg mL^{-1} of NB35 and ScFv16 at 4 °C for another 2 h. Finally, the mixture was further purified by size exclusion chromatography (SEC) using a Superdex 200 10/300 GL column (GE Healthcare) in a buffer containing 20 mM HEPES (pH 7.4), 100 mM NaCl, 0.00075% (w/v) LMNG, 0.00025% (w/v) CHS, and 1 mM spermine.

Cryo-EM grid preparation and data collection

Prior to cryo-EM grid preparation, the concentration of the spermine-bound TAAR348-Gs complex was measured by absorbance at 280 nm using a Nanodrop 2000 Spectrophotometer (Thermo Fisher) to ensure a concentration of 2.5–3.0 mg mL^{-1} in the ligand-bound state. Subsequently, 3.0 μL of the protein complex was applied to Quantifoil 300 mesh Au R1.2/1.3 grids that had been glow discharged for 45 s. The grids were blotted for 5 s under 100% humidity at 4 °C using a Mark IV Vitrobot (FEI) before being plunged into liquid ethane cooled by liquid nitrogen.

Cryo-EM imaging was performed on a Titan Krios microscope operating at 300 kV, equipped with a Gatan K3 Summit direct electron detector (Gatan) and a Gatan Quantum energy filter (operated with a slit width of 20 eV). Images were recorded in EFTEM nanoprobe mode using SerialEM software, with a 70 mm C2 aperture and a calibrated magnification of 105,000x, corresponding to a pixel size of 0.832 Å and a defocus range of −1.0 to −2.0 μm. The zero-loss peak slit width was set to 20 eV. Each movie consisted of 40 frames with a total dose of 60 e^{-} $Å^{-2}$, an exposure time of 2.0 s, and a dose rate of 20 e^{-} per pixel s^{-1}. All dose-fractionated images were first corrected for motion and dose-weighted using MotionCorr2 software, followed by estimation of their contrast transfer functions using a local CTF estimation method in cryoSPARC.

Image processing

The overall cryo-EM data processing pipeline for spermine-TAAR348-Gs is shown in Supplementary Fig. S3. All dose-fractioned images were motion-corrected and dose-weighted by MotionCorr2 software[10] and their contrast transfer functions were estimated by patch CTF estimation in cryoSPARC.[11]

A total of 5,884 movie datasets were collected for the spermine-TAAR348-Gs complex. Utilizing the automated workflow in cryoSPARC software, 123,227 particles were successfully picked from 300 images. The dataset was subjected to 2D classification into 200 classes, effectively sorting the particles. Based on this classification, 40 classes comprising 31,874 particles were selected as the training set for deep learning model training using topaz.[12] Following model training, deep recognition was performed on the 5,884 images, resulting in the automatic selection of 3,464,606 particles. After further 2D classification and screening, approximately 413,080 high-quality particles were obtained, providing a foundation for 3D reconstruction and heterogeneous refinement. During processing, C1 symmetry was applied. The final 3D heterogeneous refinement, based on 292,761 particle projections, yielded 269,228 particle projections exhibiting fine structural details. These projections underwent non-uniform refinement and local refinement, ultimately achieving a high-resolution map with a resolution of 2.82 Å. Additionally, 3D classification using a model covering the TMDs further optimized the map quality, resulting in a map with a resolution of 3.26 Å. Finally, a composite map was generated using the "vop maximum" command in UCSF Chimera, which will be utilized for subsequent model building and optimization. The overall resolution was assessed based on the gold-standard Fourier shell correlation (FSC) 0.143 criterion, while local resolution was estimated in cryoSPARC using default parameters, thereby ensuring the accuracy and reliability of the analysis results.

Model building, refinement, and validation

For the spermine-TAAR348-Gs complex, the initial model for TAAR348 was derived from the AlphaFold2 prediction of the TAAR348 from the sea lamprey. The structures of the Gs heterotrimer (comprising the Gαs, Gβ, and Gγ subunits), NB35, and ScFv16 were generated using the DMCHA-bound mTAAR9-Gs complex structure (PDB: 8ITF)[13] as a reference. The cryo-EM model was docked into the electron microscopy density map using Chimera, followed by iterative manual adjustment and rebuilding in COOT[14] to fit the cryo-EM map. The model coordinates were refined using Phenix,[15] with secondary structure and geometric restraints applied. The final structural figures were prepared using UCSF Chimera, ChimeraX, and PyMOL.

The overall structure of the spermine-TAAR348-Gs complex encompasses several key domains of the TAAR348 protein, including its seven-transmembrane domain (TMD), extracellular domain (ECD), and helix 8. Additionally, the model includes the Gs trimer (comprising the Gα, Gβ, and Gγ subunits), as well as two critical antibody components: NB35 and ScFv16. These components collectively form the three-dimensional structural framework of the complex. Notably, clear spermine density was observed within the binding pocket of TAAR348, providing insights into the binding mode of the ligand spermine.

G protein dissociation assay

The Gαs-Gβγ dissociation assay was performed as previously described.[13,16] In brief, Gαs-Nluc, Gβ3 and Gγ9-GFP were used as G protein BRET probes. HEK293 cells were transiently co-transfected with TAAR348 wild type or its mutants and G protein BRET probes. After 24 hours of transfection, HEK293 cells were distributed into 96-well microplates at a density of 5×10^4 cells/well and cultured for another 24 hours at 37 °C. Before the BRET assay, the cells were washed twice with BRET buffer (25 mM HEPES, 1 mM $CaCl_2$, 140 mM NaCl, 2.7 mM KCl, 0.9 mM $MgCl_2$, 0.37 mM NaH_2PO_4, 5.5 mM D-glucose, 12 mM $NaHCO_3$, pH=7), and then stimulated with indicated ligands at different concentrations as specified in the manuscript or figure legends. Luciferase substrate coelenterazine 400a (working concentration, 5 μM) was immediately added before reading BRET in the Mithras LB940 microplate reader (Berthold Technologies) with BRET filter sets. The BRET signal was calculated as the ratio of light emission at emitted by GFP (515 nm) and the light emitted by Nluc (400 nm). The BERT signal changes were reported as ΔBRET.

Enzyme-linked immunosorbent assay

As previously described,[17] to monitor the expression levels of the TAAR348 wild type or mutant proteins before the BRET assay, the TAAR348-plasmid-transfected cells were seeded into 96-well plates and cultured at 37 °C for 24 h. The cells were washed three times with PBS, fixed by 4% (w/v) paraformaldehyde for 5 minutes at room temperature, blocked with 5% (w/v) BSA for 1 h and then probed with anti-Flag antibodies (Sigma-Aldrich, 1 : 1000) for 12 h at 4 °C. These cells were washed and incubated with secondary anti-mouse antibodies (Thermo

Fisher, 1 : 5000). After washing, TMB substrate (Milllipore) was added for color reaction, and were stopped with 0.25 M HCl. The results were analyzed with a TECAN luminescence counter (Infinite M200 Pro Nano Quant) at a wavelength of 450 nm. The expression levels of the mutants were normalized to that of the wild-type TAAR348. Detailed results are presented in Supplementary Fig. S8e and Table S3.

WebLogo of TAAR

A total of 25 TAARs, including TAAR348, TAAR1, TAAR5, TAAR6, TAAR7f, TAAR8, TAAR9, TAAR13c, and HTR4, were collected from human, mouse, rat, and zebrafish. The sequences were aligned using the L-INS-i,[1] and sequence logos were generated using WebLogo 3.7.12 based on pocket site residues.[18] The color scheme was applied using the -c chemistry, which colors residues based on their chemical properties. RMSD plot of spermine binding in the TAAR348 pocket compared to the cryo-EM structure during molecular dynamics simulations. Detailed results are presented in Supplementary Fig. S8a.

Molecular dynamics simulation

The spermine-TAAR348-Gs simulation models were constructed based on the ligand-bound cryo-EM structure described in this manuscript. Protein processing was conducted using the Protein Preparation workflow tool in Maestro software of the Schrodinger platform v2022-3.[19] In this step, hydrogen atoms were added, missing sidechain atoms were filled, and ACE (N-acetyl) and NMA (N-methyl amide) groups were appended to uncapped N and C termini. The spermine was protonated to correspond to the dominant protonation state at pH 7.0 ± 2.0, all N are charged except for the N at the bottom of the pocket, facilitating the formation of the conserved salt bridge with the neighboring D112$^{3.32}$, E277$^{6.58}$. Prepared protein structures were aligned on the transmembrane helices to the Orientation of Proteins in Membranes (OPM).[20] Subsequently, the structures were embedded into a palmitoyl-oleoyl-phosphatidylcholine (POPC) bilayer and the system was neutralized by adding 0.15 mM NaCl. The CHARMM36m parameter set was employed for protein molecules, lipid molecules and salt ions, while the CHARMM TIP3P model was used for water.[21] Parameters for spermine was generated using the CHARMM General Force Field (CGenFF).[22]

MD simulations were conducted using GROMACS 2023.3.[23] Each system was replicated to perform three independent simulations. For each system, the steepest descent algorithm was employed until the maximum force was smaller than 1,000 kJ mol^{-1}·nm^{-1}. Prepared systems were minimized, then equilibrated as follows: The system was initially heated from 0 to 100 K using the V-rescale method in the NVT ensemble over 100 ps, followed by heating to 310 K over 1000 ps. Harmonic restraints of 1000 kJ mol^{-1}·nm^{-2} were applied to the non-hydrogen atoms of lipid, protein, and ligand, and initial velocities were sampled from the Maxwell distribution. Subsequently, the system was equilibrated with position restraints on non-hydrogen atoms of protein and ligand in the NPT ensemble with isotropic pressure coupling at a pressure of one bar. This was done first with a force constant of 1,000 kJ mol^{-1}·nm^{-2}, and then gradually reducing the restraint force following 800, 600, 400, and 200 kJ mol^{-1}·nm^{-2}, each for 1 ns. Finally, productive simulations were conducted in the NPT ensemble with semi-isotropic pressure coupling at a pressure of one bar for 500 ns. The equations of motion were integrated using a 2-fs time step, with the leap-frog algorithm employed as the integrator. Constraints were applied to the bonds with hydrogen atoms using the LINCS method. The cut-off distance for Van der Waals and short-range electrostatic interactions was set to 10 Å, and long-range electrostatic interactions were computed using the smooth particle mesh Ewald method.

Trajectory snapshots were saved every 200 ps during production simulations. The root-mean-square deviation (RMSD) of spermine was calculated relative to the crystal structure.

a

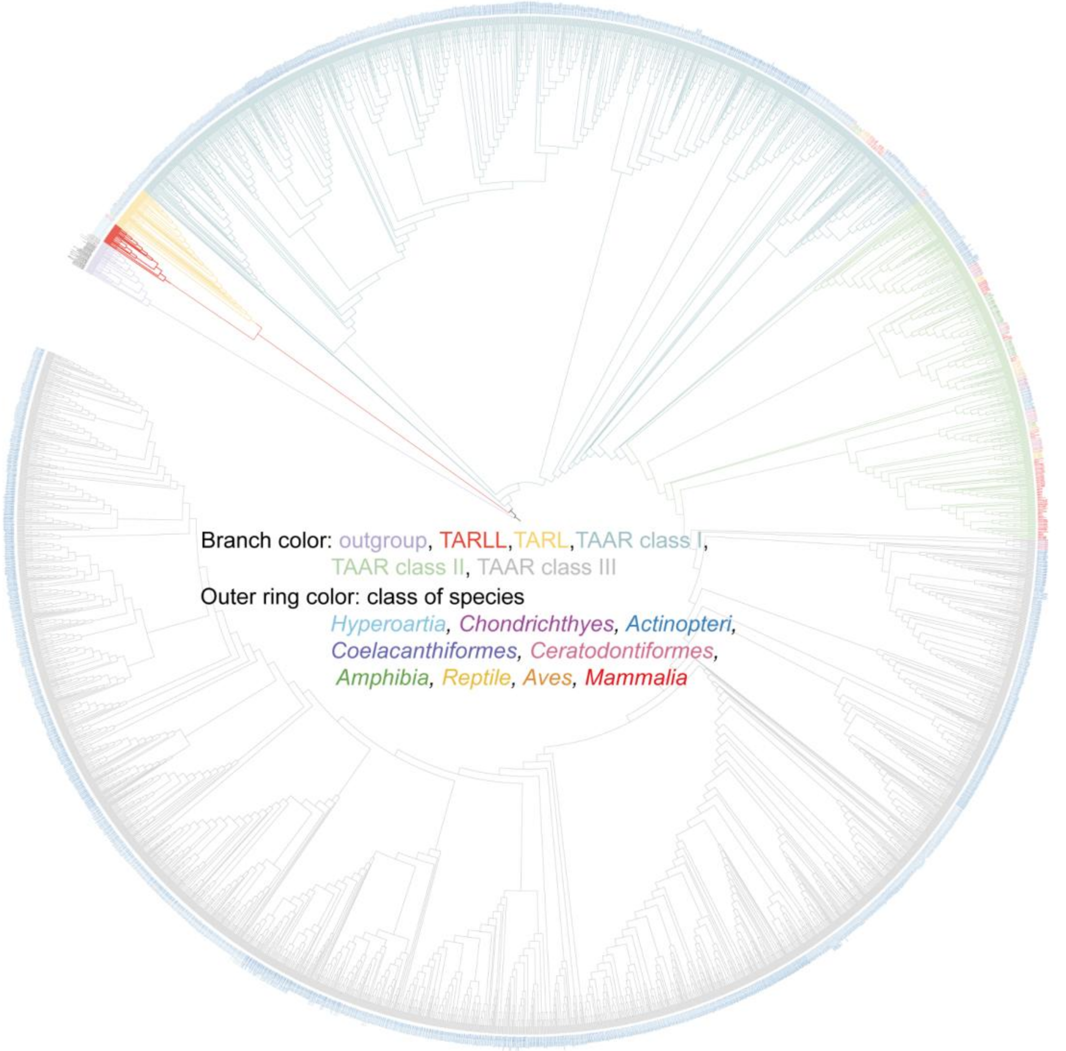

b

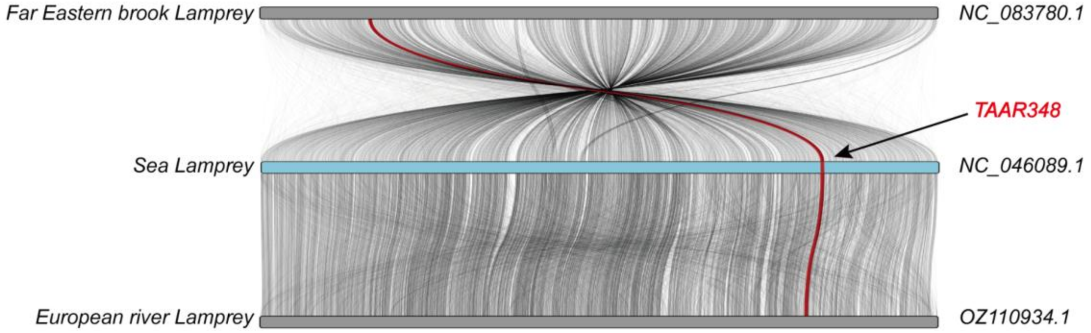

Supplementary Fig. S1 | Phylogenetic and genomic analysis of TAAR348 and its orthologues across vertebrates

(a) Phylogenetic Analysis of TAAR in Chordates. TARLL is exclusively present in jawless fish (Hyperoartia), which lack other TAAR subtypes. In contrast, other aminergic receptors, TARL, TAAR class I, TAAR class II, and TAAR class III, exhibit a broad taxonomic distribution across diverse species. A maximum-likelihood (ML) phylogenetic tree of

TAAR protein sequences was constructed using IQ-TREE under the JTT+F+R5 model. The tree was visualized with iTOL. The outer ring is color-coded according to species classification, while branch colors represent different TAAR classes.

(b) The pairwise genome alignments reveal the chromosomal region harboring TAAR348 and its one-to-one orthologs. Gray lines represent all syntenic blocks shared between the Far Eastern brook lamprey, European river lamprey, and Sea lamprey, while the red line highlights the conserved genomic position of TAAR348 and its orthologs across these three lamprey species. Alignment results demonstrated strong conservation of the TAAR348-containing chromosomal region across the three lamprey species. Notably, a chromosomal inversion was observed in the Far Eastern brook lamprey (NC_083780.1) relative to the reference genome (Sea lamprey, NC_046089.1).

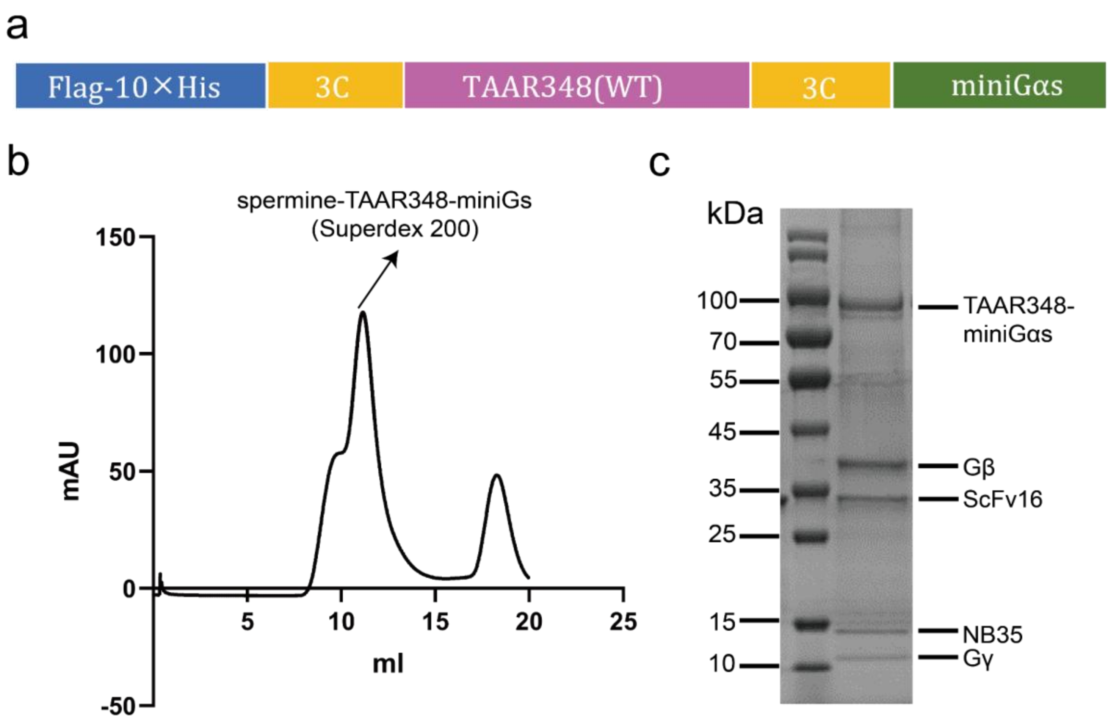

Supplementary Fig. S2 | Construction and purification of the spermine-bound TAAR348-Gs complex

(a) Cartoon model of the TAAR348 construct used in this study.

(b-c) Representative size exclusion chromatography (SEC) profiles and SDS-PAGE analysis of the spermine-TAAR348-Gs complex. The experiment was repeated at least three times with similar results.

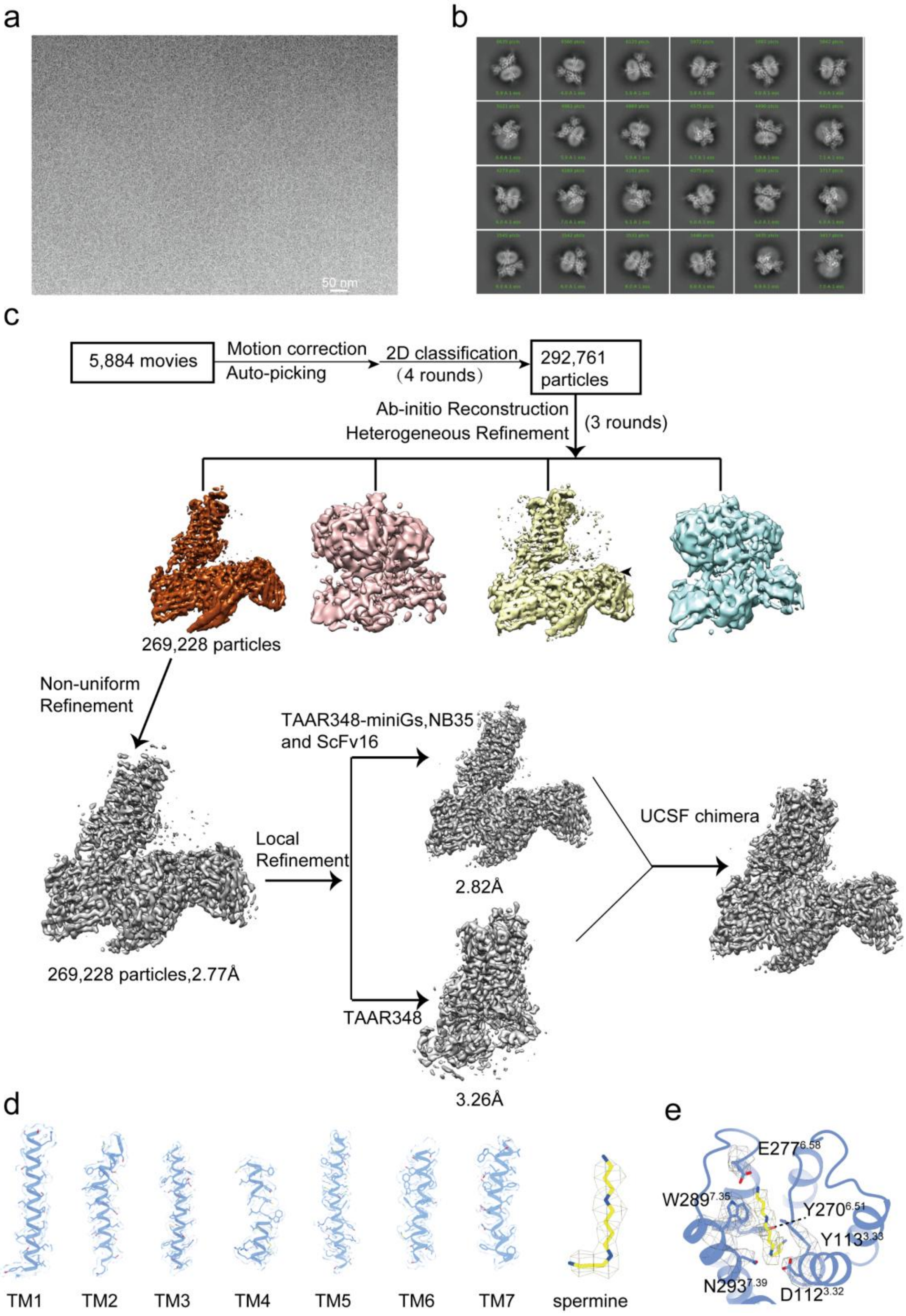

Supplementary Fig. S3 | Structure determination of spermine-bound TAAR348-Gs complex

(a-b) Representative cryo-EM image (a) and 2D classification averages (b) of spermine-TAAR348-Gs complex.

(c) Cryo-EM data processing flowchart of spermine-TAAR348-Gs complex by cyroSPARC 3.2.

(d) Cryo-EM density maps of the transmembrane helices TM1-TM7 of TAAR348, and spermine in the spermine-TAAR348-Gs complex.

(e) Cryo-EM density maps of spermine within the TAAR348 binding pocket and the surrounding key residues. The density map is shown at a contour level of 0.181.

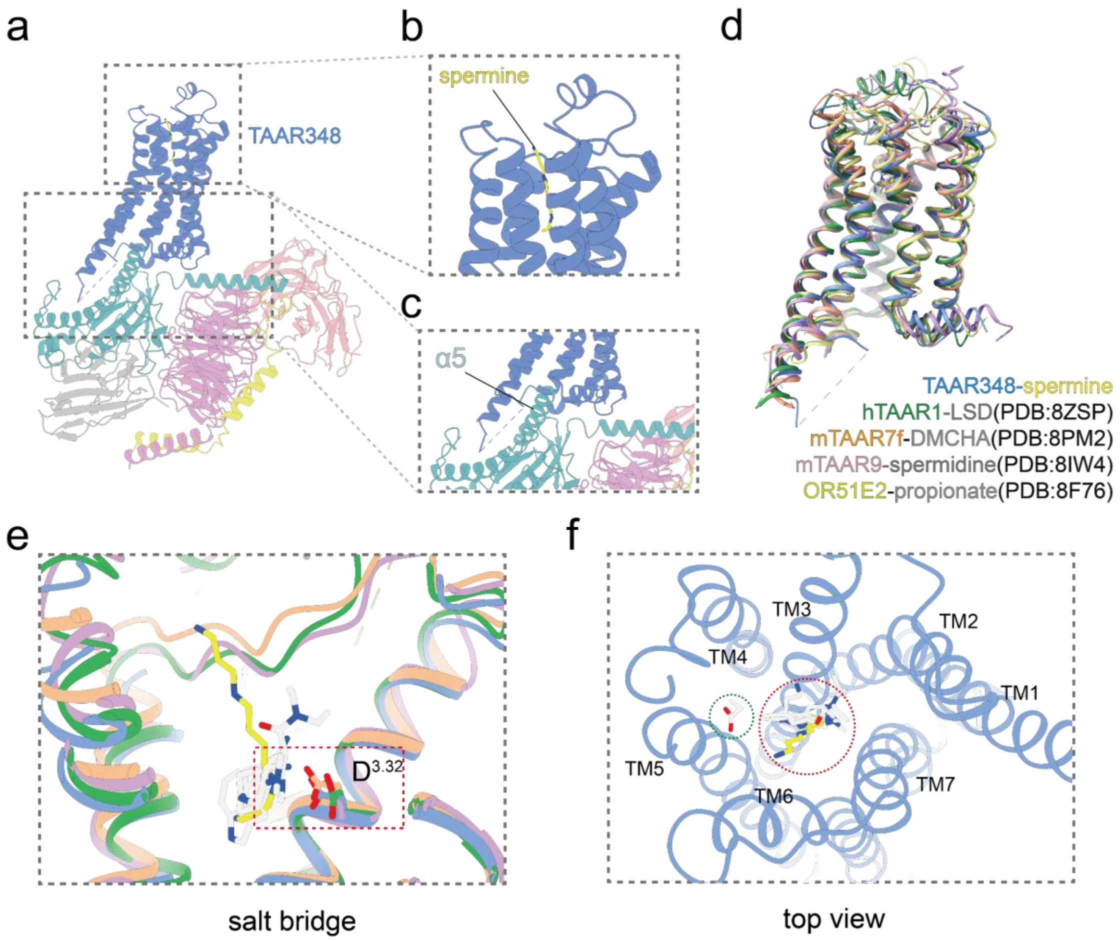

Supplementary Fig. S4 | Characteristics of TAAR348 structure and comparative analysis with other TAARs and olfactory receptors

(a-c) Cartoon representations of the spermine-TAAR348-Gs complex. Panels (b) and (c) show enlarged views of specific regions.

(d) Structural alignment of spermine-bound TAAR348 with other TAARs bound to different ligands and the canonical olfactory receptor OR51E2.

(e) Various ligands form salt bridges with the conserved aspartate residue $D^{3.32}$ in TAARs.

(f) Structural alignment of ligand-bound TAARs and propionic acid (green circle)-bound canonical olfactory receptor OR51E2, highlighting the different positions of their binding pockets.

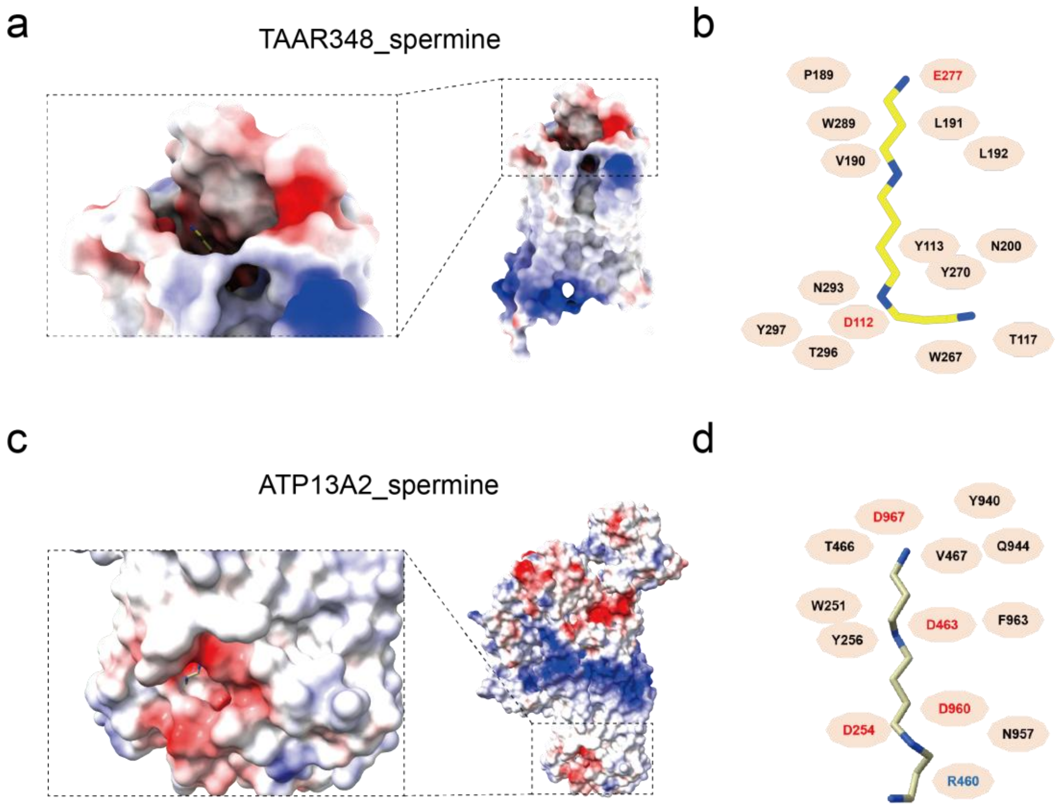

Supplementary Fig. S5 | Spermine recognition: comparison of electrostatic potential and residue charge properties in the binding pockets of TAAR348 and ATP13E2

(a) Electrostatic potential map of the spermine pocket in TAAR348. Red regions indicate negative electrostatic potential, demonstrating that the spermine pocket is predominantly negatively charged.

(b) Residues surrounding the spermine pocket in TAAR348 are displayed. Negatively charged residues are indicated in red, highlighting the prevalence of negatively charged residues in this region.

(c) Electrostatic potential map of the spermine pocket in ATP13E2. Red regions indicate negative electrostatic potential, showing that the spermine pocket is also predominantly negatively charged.

(d) Residues surrounding the spermine pocket in ATP13E2 are displayed. Negatively charged residues are indicated in red, while positively charged residues are shown in blue, highlighting the prevalence of negatively charged residues in this region.

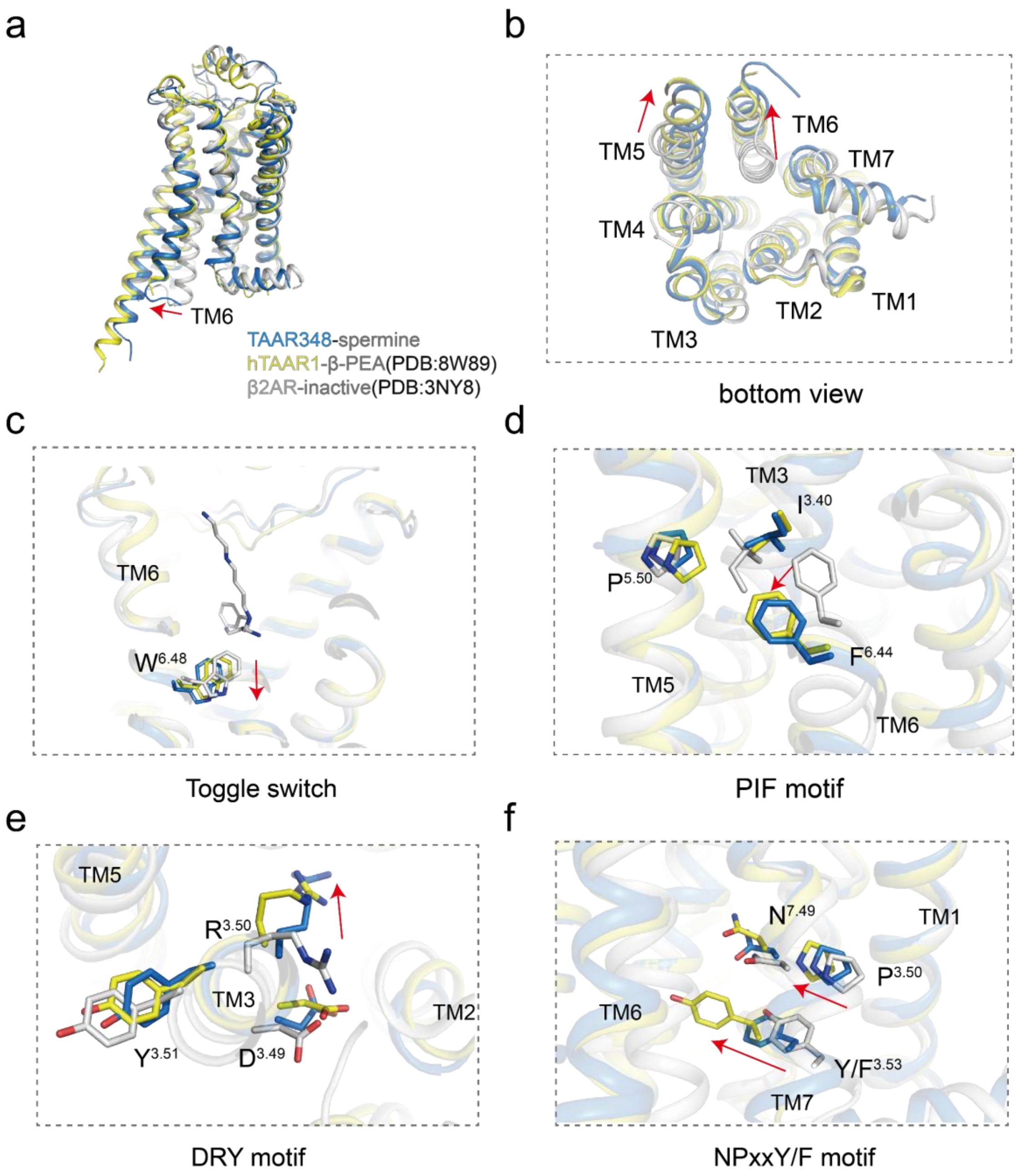

Supplementary Fig. S6 | Activation motif of TAAR348

(a-f) Close-up views of activation-related conformational changes in key motifs that show the connection of structural changes from the orthosteric binding site to the cytoplasmic transducer binding site. Notable conformational changes occur at intracellular ends of TM5 and TM6 upon receptor activation, bottom view **(b)**. Conformational changes between TAAR348 (blue) in spermine-bound state, active TAAR1 (yellow), and β2AR (gray) in inactive state (PDB: 3NY8) are highlighted. Activation switch $W^{6.48}$ **(c)**, PIF motif **(d)**, DRY motif **(e)**, and NPxxY/F motif **(f)**.

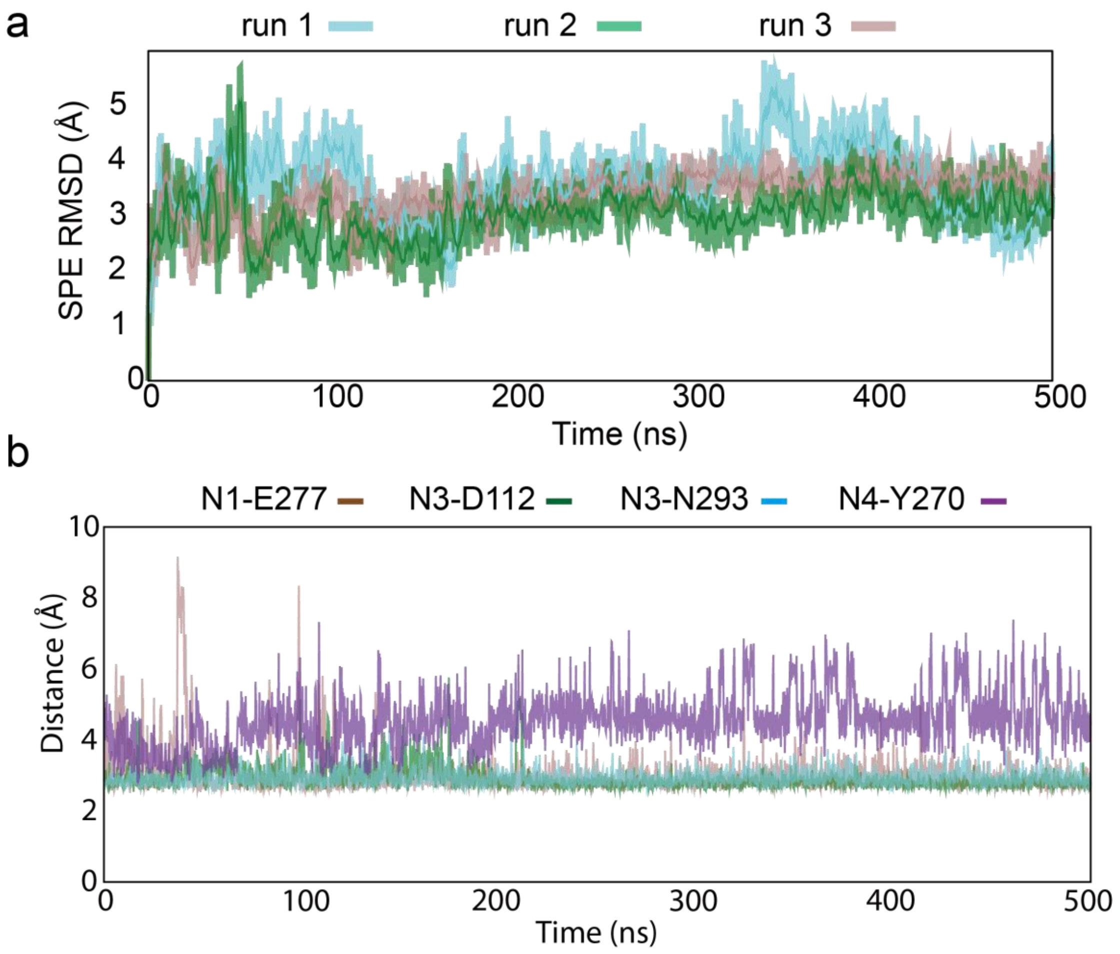

Supplementary Fig. S7 | MD-simulation analysis of the TAAR348–spermine complex

(a) RMSD plot of spermine binding in the TAAR348 pocket within 500 ns of simulations.

(b) Distance between the N atom of spermine and the hydrogen-bonding atom (or OH group) of the paired residue in run 3 MD simulation.

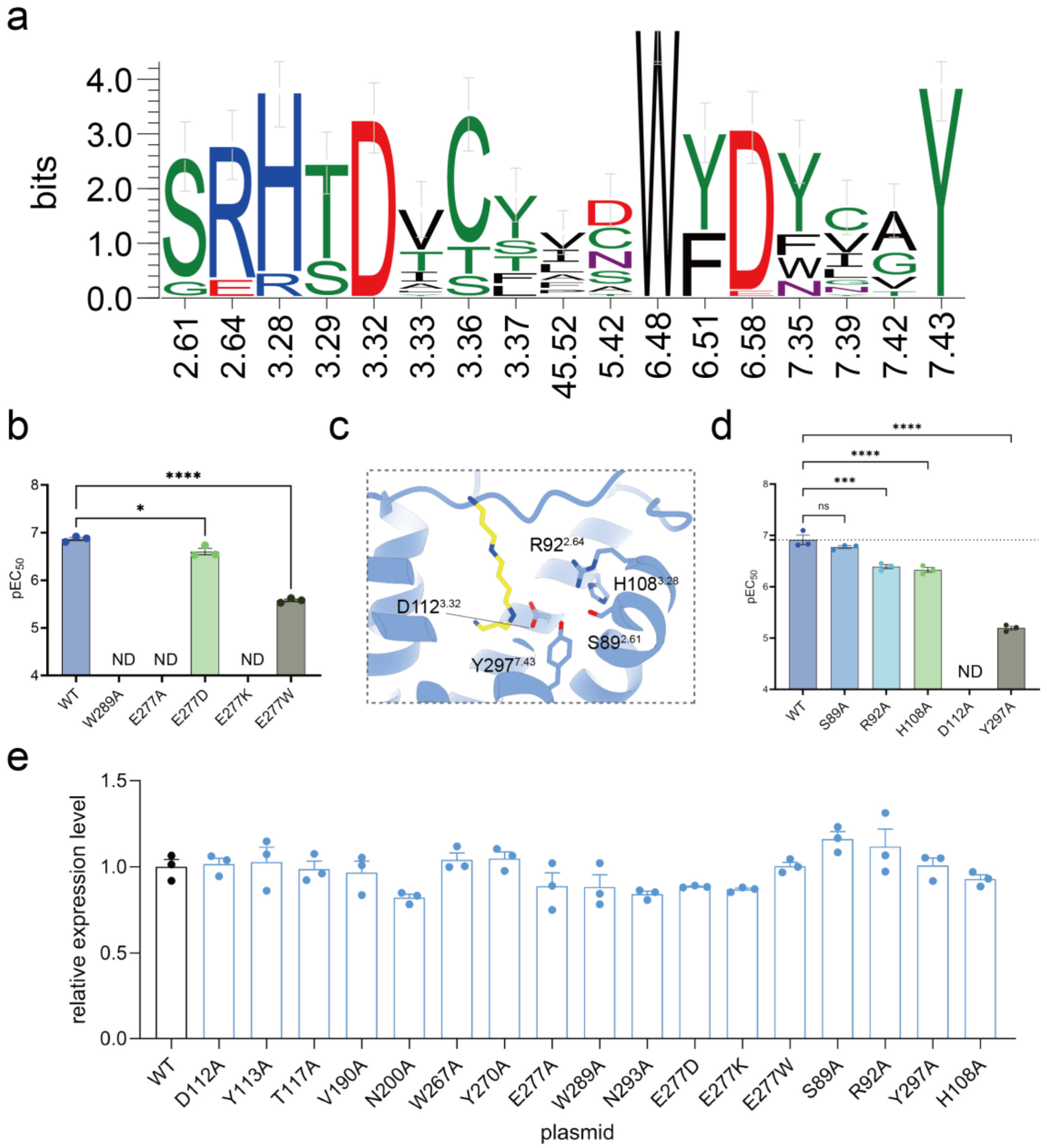

Supplementary Fig. S8 | Functional analysis to elucidate TAAR348's amine recognition mechanism

(a) WebLogo of TAAR. Sequence logos for 25 TAARs from human, mouse, rat, and zebrafish, generated using WebLogo 3.7.12 with -c chemistry coloring.

(b) The bar chart shows the potency of ligand binding to TAAR348 mutants, measured by pEC_{50} values obtained from BRET2 assays in HEK293 cells overexpressing TAAR348 proteins. The pEC_{50} values are presented as the negative logarithm of the half-maximal effective concentration. The mutants analyzed include various amino acid substitutions at the $E^{6.58}$-$W^{7.35}$ motif (primarily $E^{6.58}$) with different physicochemical properties. Error bars

represent the SEM from three independent experiments ($n = 3$). Statistical significance compared to the wild-type receptor was determined using a two-sided Welch's t-test (****$p < 0.0001$; **$p = 0.001$ to 0.01; *$p = 0.01$ to 0.05; ns not significant, $p \geq 0.05$). ND, signal not detectable.

(c) Polar interaction network surrounding the conserved aspartate residue $D^{3.32}$ in spermine-bound TAAR348.

(d) Ligand binding potency to TAAR348 mutants. Bar chart showing the potency of ligand binding to TAAR348 mutants with alanine substitutions within the polar network. Assays performed as in **(b)**.

(e) ELISA data summarizing the expression levels of wild type and mutants of TAAR348. Values are represented as mean ± SEM of 3 independent experiments ($n = 3$). n.s., not significant. All data were analyzed by two-sided one-way ANOVA with Tukey's test.

Supplementary Table S1 | Reference information of species that were employed in orthologues alignment

Seq	Class	Family	Scientific_name	Common_name	Accession	Version	Level	N50
1	Jawless	Petromyzontidae	Petromyzon marinus	sea lamprey	GCF_010993605.1	kPetMar1.pri	Chromosome	13
2	Jawless	Petromyzontidae	Lampetra fluviatilis	European river lamprey	GCA_964198595.1	kcLamFluv1.1	Chromosome	13.2
3	Jawless	Petromyzontidae	Lethenteron reissneri	Far Eastern brook lamprey	GCF_015708825.1	ASM1570882v1	Chromosome	13.5
4	Jawless	Myxinidae	Eptatretus atami	brown hagfish	GCA_035128595.1	Eptata_v1	Chromosome	147.6
5	Jawless	Myxinidae	Myxine glutinosa	Atlantic hagfish	GCA_964187855.1	kmMyxGlut1.1	Chromosome	192.2
6	Jawed	Chondrichthyes	Amblyraja radiata	thorny skate	GCF_010909765.2	sAmbRad1.1.pri	Chromosome	62.1
7	Jawed	Chondrichthyes	Mobula hypostoma	lesser devil ray	GCF_963921235.1	sMobHyp1.1	Chromosome	152.4
8	Jawed	Teleostomi	Polypterus senegalus	gray bichir	GCF_016835505.1	ASM1683550v1	Chromosome	189.7
9	Jawed	Teleostomi	Danio rerio	zebrafish	GCF_000002035.6	GRCz11	Chromosome	7.4
10	Jawed	Teleostomi	Protopterus annectens	West African lungfish	GCF_019279795.1	PAN1.0	Chromosome	2048

Supplementary Table S2 | Cryo-EM data collection, refinement, and validation statistics

	spermine-TAAR348-Gs
PDB	9VMG
EMDB	EMD-65165<br>EMD-65169<br>EMD-65187
**Data collection and processing**	
Magnification	105,000
Voltage (kV)	300 kV
Pixel size (Å)	0.832
Electron exposure (e^{-}/Å^2)	~60
Defocus range (μm)	-1.0 to -2.0
Symmetry imposed	C1
Final particle images (no.)	269,228
Map resolution (Å)<br>FSC threshold	2.82<br>0.143
**Refinement**	
Initial model used<br>(PDB code)	AlphaFold2<br>8ITF
Map sharpening *B*-factor (Å^2)	N/A
Model composition	
Non-hydrogen atoms<br>Protein residues<br>Ligands	9818<br>1270<br>spermine
R.m.s. deviations	
Bond lengths (Å)<br>Bond angles (°)	0.004<br>0.609
Validation	
MolProbity score<br>Clash score<br>Poor rotamers (%)	1.79<br>10.69<br>0.10
Ramachandran plot	
Favored (%)<br>Allowed (%)<br>Disallowed (%)	96.33<br>3.67<br>0.0

Supplementary Table S3 | Cell surface expression of TAAR348 and its mutants

Mean is defined as percent WT maximum response. Data represent mean ± SEM of n = 3 biological replicate.

	**Mean ± SEM (% TAAR348-wt)**
**TAAR348-wt**	100.00±4.30
**D112A**	101.55±3.55
**Y113A**	102.82±8.57
**T117A**	98.71+4.58
**V190A**	96.70±6.63
**N200A**	82.20±2.02
**W267A**	104.10±3.90
**Y270A**	104.86±3.77
**E277A**	88.76±7.84
**W289A**	88.28±7.16
**N293A**	84.19±1.71
**E277D**	88.56±0.31
**E277K**	86.88±0.80
**E277W**	100.47±2.26
**S89A**	116.11±4.28
**R92A**	111.76±10.14
**Y297A**	100.75±4.47
**H108A**	93.00±2.43

References

1 Nakamura, T., Yamada, K. D., Tomii, K. & Katoh, K. Parallelization of MAFFT for large-scale multiple sequence alignments. *Bioinformatics* **34**, 2490-2492 (2018).

2 Minh, B. Q. *et al.* Corrigendum to: IQ-TREE 2: New Models and Efficient Methods for Phylogenetic Inference in the Genomic Era. *Mol Biol Evol* **37**, 2461 (2020).

3 Letunic, I. & Bork, P. Interactive Tree of Life (iTOL) v6: recent updates to the phylogenetic tree display and annotation tool. *Nucleic Acids Res* **52**, W78-W82 (2024).

4 Fu, L., Niu, B., Zhu, Z., Wu, S. & Li, W. CD-HIT: accelerated for clustering the next-generation sequencing data. *Bioinformatics* **28**, 3150-3152 (2012).

5 UniProt, C. UniProt: the Universal Protein Knowledgebase in 2025. *Nucleic Acids Res* **53**, D609-D617 (2025).

6 Guo, L. *et al.* Evolution of Brain-Expressed Biogenic Amine Receptors into Olfactory Trace Amine-Associated Receptors. *Mol Biol Evol* **39** (2022).

7 Kumar, S. *et al.* TimeTree 5: An Expanded Resource for Species Divergence Times. *Mol Biol Evol* **39** (2022).

8 Chun, E. *et al.* Fusion partner toolchest for the stabilization and crystallization of G protein-coupled receptors. *Structure* **20**, 967-976 (2012).

9 Barak, L. S. *et al.* Pharmacological characterization of membrane-expressed human trace amine-associated receptor 1 (TAAR1) by a bioluminescence resonance energy transfer cAMP biosensor. *Mol Pharmacol* **74**, 585-594 (2008).

10 Zheng, S. Q. *et al.* MotionCor2: anisotropic correction of beam-induced motion for improved cryo-electron microscopy. *Nat Methods* **14**, 331-332 (2017).

11 Punjani, A., Rubinstein, J. L., Fleet, D. J. & Brubaker, M. A. cryoSPARC: algorithms for rapid unsupervised cryo-EM structure determination. *Nat Methods* **14**, 290-296 (2017).

12 Bepler, T. *et al.* Positive-unlabeled convolutional neural networks for particle picking in cryo-electron micrographs. *Nat Methods* **16**, 1153-1160 (2019).

13 Guo, L. *et al.* Structural basis of amine odorant perception by a mammal olfactory receptor. *Nature* **618**, 193-200 (2023).

14 Emsley, P. & Cowtan, K. Coot: model-building tools for molecular graphics. *Acta Crystallogr D Biol Crystallogr* **60**, 2126-2132 (2004).

15 Adams, P. D. *et al.* PHENIX: a comprehensive Python-based system for macromolecular structure solution. *Acta Crystallogr D Biol Crystallogr* **66**, 213-221 (2010).

16 Yang, Z. *et al.* Identification, structure, and agonist design of an androgen membrane receptor. *Cell* **188**, 1589-1604 e1524 (2025).

17 Shang, P. *et al.* Structural and signaling mechanisms of TAAR1 enabled preferential agonist design. *Cell* **186**, 5347-5362.e5324 (2023).

18 Crooks, G. E., Hon, G., Chandonia, J. M. & Brenner, S. E. WebLogo: a sequence logo generator. *Genome Res* **14**, 1188-1190 (2004).

19 Sastry, G. M., Adzhigirey, M., Day, T., Annabhimoju, R. & Sherman, W. Protein and ligand preparation: parameters, protocols, and influence on virtual screening enrichments. *J Comput Aided Mol Des* **27**, 221-234 (2013).

20 Lomize, M. A., Lomize, A. L., Pogozheva, I. D. & Mosberg, H. I. OPM: orientations

of proteins in membranes database. *Bioinformatics* **22**, 623-625 (2006).

21 Huang, J. *et al.* CHARMM36m: an improved force field for folded and intrinsically disordered proteins. *Nat Methods* **14**, 71-73 (2017).

22 Vanommeslaeghe, K. *et al.* CHARMM general force field: A force field for drug-like molecules compatible with the CHARMM all-atom additive biological force fields. *J Comput Chem* **31**, 671-690 (2010).

23 Abraham, M. J. *et al.* GROMACS: High performance molecular simulations through multi-level parallelism from laptops to supercomputers. *SoftwareX* **1-2**, 19-25 (2015).
